# Anticoagulant and Antithrombotic Properties in Vitro and in Vivo of a Novel Sulfated Polysaccharide from Marine Green Alga *Monostroma nitidum*

**DOI:** 10.3390/md17040247

**Published:** 2019-04-25

**Authors:** Sujian Cao, Xiaoxi He, Ling Qin, Meijia He, Yajing Yang, Zhichun Liu, Wenjun Mao

**Affiliations:** 1 Key Laboratory of Marine Drugs of Ministry of Education, Shandong Provincial Key Laboratory of Glycoscience and Glycotechnology, School of Medicine and Pharmacy, Ocean University of China, Qingdao 266003, China; caosujian@stu.ouc.edu.cn (S.C.); hexiaoxi@ouc.edu.cn (X.H.); ql1599@stu.ouc.edu.cn (L.Q.); hmj@stu.ouc.edu.cn (M.H.); yangyajing@stu.ouc.edu.cn (Y.Y.); liuzhichun@stu.ouc.edu.cn (Z.L.); 2 Laboratory for Marine Drugs and Bioproducts of Qingdao National Laboratory for Marine Science and Technology, Qingdao 266237, China

**Keywords:** marine green algae, sulfated polysaccharide, structural characteristics, anticoagulant property, antithrombotic activity

## Abstract

Sulfated polysaccharides from marine algae have high potential as promising candidates for marine drug development. In this study, a homogeneous sulfated polysaccharide from the marine green alga *Monostroma nitidum*, designated MS-1, was isolated using water extraction and anion-exchange and size-exclusion chromatography. Results of chemical and spectroscopic analyses showed that MS-1 mainly consisted of →3)-α-l-Rha*p*-(1→ and →2)-α-l-Rha*p*-(1→ residues, with additional branches consisting of 4-linked β-d-xylose, 4-/6-linked d-glucose, terminal β-d-glucuronic acid, and 3-/2-linked α-l-rhamnose. Sulfate ester groups substituted mainly at C-2/C-4 of →3)-α-l-Rha*p*-(1→ and C-4 of →2)-α-l-Rha*p*-(1→ residues, slightly at C-2 of terminal β-d-glucuronic residues. MS-1 exhibited strong anticoagulant activity in vitro and in vivo as evaluated by the activated partial thromboplastin time and thrombin time assays, and significantly decreased platelet aggregation. The anticoagulant activity mechanism of MS-1 was mainly attributed to strong potentiation thrombin by heparin cofactor-II, and it also hastened thrombin and coagulation factor Xa inhibitions by potentiating antithrombin-III. MS-1 possessed markedly thrombolytic activity evaluated by plasminogen activator inhibitior-1, fibrin degradation products, and D-dimer levels using rats plasma, and recanalization rate by FeCl_3_-induced carotid artery thrombosis in mice. MS-1 exhibited strong antithrombotic activity in vitro and in vivo evaluated by the wet weighs and lengths of thrombus, and thrombus occlusion time by electrically-induced carotid artery thrombosis in rats. These results suggested that MS-1 could be a promising marine drug for prevention and therapy of thromboembolic disease.

## 1. Introduction

Thrombus usually occurs in vessels with severe atherosclerotic disease and may embolize to cause transient ischemic attacks and cerebral infarctions [1]. Thrombosis is a result of an activation process of thrombin that converts fibrinogen into fibrin, eventually resulting in insoluble fibrin polymer or clot [2]. The Global Burden of Disease Study 2010 reported that the ischemic heart disease and stroke collectively caused one in four deaths worldwide [3]. Anticoagulant drugs have been extensively used as an adjunct therapy in thrombotic diseases, and heparin is one of the agents more widely used in the therapy and prophylaxis of thrombosis. However heparin is mostly extracted from pig intestine or bovine lung and the tissues are rich in mast cells, presumably resulting from the high foreign parasite burden in the tissues. The incidence of prion-related diseases in mammals and the appearance of ‘mad cow disease’ have limited the use of animal heparin. Furthermore, heparin is associated with various side effects [4,5]. Therefore, new instead sources of heparin are urgently desired [6].

Marine algae accumulate large amounts of sulfated polysaccharides in their cell walls or intercellular regions [7,8]. These sulfated polysaccharides exhibit various biological activities, including anticoagulant, antihyperlipidemic, immunomodulatory, and antiviral activities [9,10,11]. So far, algal sulfated polysaccharides are only partly explored, especially the sulfated polysaccharides from marine green algae. Some investigations revealed that the sulfated polysaccharides from marine green algae were one abundant and unique source of heparinoids, and had high potential as preventing and therapeutic agents against thrombotic diseases [12,13,14,15,16,17,18].

The marine green alga *Monostroma nitidum* is widely distributed through the world’s seas, and has been cultivated as edible alga in some countries. In the present study, a novel sulfated polysaccharide was obtained from *M. nitidum*. The structural characteristics, anticoagulant and antithrombotic properties in vitro and in vivo of the sulfated polysaccharide were investigated. The sulfated polysaccharide has promising as a marine drug for thrombotic disease. The results of our investigation are given in the paper.

## 2. Results and Discussion

### 2.1. Isolation and Chemical Composition of MS-1

The crude polysaccharide was extracted by hot water and then fractionated using a Q Sepharose Fast Flow column into two fractions. The fraction eluted with 0.5 mol/L NaCl contained the most abundant total sugar and was then further purified by Sephacryl S-400/HR column. Finally, one purified Monostroma sulfated polysaccharide (MS-1) was obtained. The yield of MS-1 from starting material was about 1.34% (w/w).

MS-1 was a homogenous polysaccharide due to it appeared as a single and symmetrical peak on the high performance gel permeation chromatography (HPGPC) (Figure S1a). The molecular weight of MS-1 was ~79.8 kDa based on the calibration graph prepared with pullulan standards. The monosaccharide composition analysis (Figure S1b) showed that MS-1 consisted of rhamnose, glucuronic acid, glucose, and xylose in the molar ratio of 35.41:1.28:1.36:1.00. The sugar configuration analysis showed that rhamnose was the l-configuration, whereas glucuronic acid, xylose, and glucose were the d-configuration (Figure S1c). The sulfate ester content of MS-1 was 28.20% and the glucuronic acid was 3.81%. No protein was found in MS-1.

Fourier-transform infrared (FTIR) spectrum of MS-1 was shown in Figure S1d. The characteristic band at 3400 cm^−1^ was attributed to the O–H stretching vibrations, and the absorption band at 2930 cm^−1^ was due to the C–H stretching vibration. The band at 1051 cm^−1^ was from the stretching vibration of C–O and change angle vibration of O–H. Furthermore, the band at 1460 cm^−1^ could be the stretching vibration of the carbonyl group. The absorption peaks at 850 and 1240 cm^−1^ derived from the stretching vibration of C–O–S of sulfate ester in axial position and S–O stretching vibration, respectively.

### 2.2. Structural Characteristics of MS-1

#### 2.2.1. Methylation Analysis

In order to obtain the information of the linkage pattern and the sulfate ester position in MS-1, a comparative methylation analysis between the native MS-1 and its desulfated polysaccharide product (DSMS-1) was carried out. As shown in Table 1, MS-1 was mainly composed of →3)-Rha*p*-(1→, →2)-Rha*p*-(1→ and →2,3)-Rha*p*-(1→ residues, followed by →3,4)-Rha*p*-(1→ and →2,4)-Rha*p*-(1→ residues. Compared with the result of methylation analysis of DSMS-1, the →3)-Rha*p*-(1→ and →2)-Rha*p*-(1→ residues were significantly increased, whereas the amounts of →2,3)-Rha*p*-(1→, →2,4)-Rha*p*-(1→ and →3,4)-Rha*p*-(1→ residues decreased. Moreover, no →4)-Rha*p*-(1→ residue was found in DSMS-1, with the disappearance of →2,4)-Rha*p*-(1→ and →3,4)-Rha*p*-(1→ residues. Thus, the sulfate ester groups substituted at the C-4/C-2 of →3)-Rha*p*-(1→ and C-4 of →2)-Rha*p*-(1→ residues. In addition, minor amounts of Xyl*p*-(1→, Glc*p*-(1→, →4)-Xyl*p*-(1→, →4)-Glc*p*-(1→ and →6)-Glc*p*-(1→ residues were detected in MS-1 and DSMS-1. Moreover it could be deduced that ~23.31% of the rhamnose residues in MS-1 were substituted by sulfate ester groups, especially for →3)-Rha*p*-(1→ residue was about 15.15%, for →2)-Rha*p*-(1→ residue was about 8.16%. 

#### 2.2.2. NMR Spectroscopy Analysis of DSMS-1 

**Figure 1:**
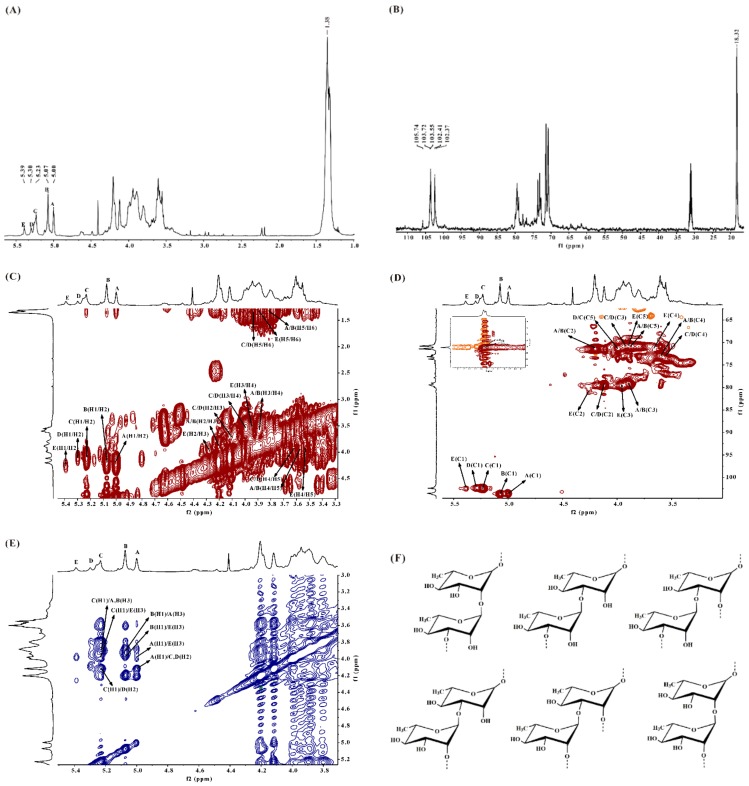
NMR spectra and structures of the main repeating disaccharides of DSMS-1. Spectra were performed on an Agilent DD2 500M NMR spectrometer. Chemical shifts are referenced to internal acetone at 2.225 ppm for ^1^H and 31.07 ppm for ^13^C. (**A**) ^1^H NMR spectrum, (**B**) ^13^C NMR spectrum, (**C**) ^1^H–^1^H COSY spectrum, (**D**) ^1^H–^13^C HSQC spectrum, (**E**) ^1^H–^1^H NOESY spectrum, and (**F**) structures of the main repeating disaccharides in DSMS-1. A–E correspond to →3)-α-l-Rha*p*-(1→, →3)-α-l-Rha*p*-(1→, →2)-α-l-Rha*p*-(1→, →2)-α-l-Rha*p*-(1→ and →2,3)-α-l-Rha*p*-(1→, respectively. Rha*p*: rhamnopyranose.

In the ^1^H NMR spectrum of DSMS-1 (Figure 1A), there were three main anomeric signals at 5.00 (A), 5.07 (B), and 5.23 (C) ppm, and two relatively weak signals at 5.29 (D) and 5.39 (E) ppm. The relative integrals of A, B, C, D, and E were 1.00:1.43:1.24:0.38:0.60. The five anomeric proton signals were assigned to the α-l-rhamnopyranose residues. The signal at 1.38 ppm was the proton of CH_3_ group of rhamnose residues. In the ^13^C NMR spectrum (Figure 1B), the anomeric carbon signals at 102.40~103.70 ppm were assigned to α-l-rhamnopyranose units (compared with that of β-anomer at 105 ppm) [19]. The signal at 18.32 ppm was assigned to C-6 of the rhamnose residues. Other signals at 70–80 ppm were attributed to C-2–C-5 of the rhamnose residues. The α-anomeric configuration of rhamnosyl units was also deduced from H-5 signals at 3.91–3.98 ppm (compared with the β-anomer at 3.39 ppm) and C-5 signal at 70.73 ppm (compared with that of the β-anomer at 72.3–73.4 ppm). Due to the relatively low contents of glucuronic acid, xylose, and glucose and overlapping of signals, it was very difficult to identify their chemical shifts. However, the low intensity signal at 105.74 ppm could be from β-d-glucuronic acid and β-d-xylosyl units [20].

The ^1^H NMR spin systems of the polysaccharide were assigned by the ^1^H–^1^H COSY spectrum (Figure 1C). The direct C–H coupling was determined by the ^1^H–^13^C HSQC spectrum (Figure 1D). Combined with the analysis of the ^1^H–^13^C HSQC spectrum and the comparison with the chemical shift data of similarly substituted sugar residues, the assignment of the main signals of the five sugar residues could be completed. The anomeric proton signals of A and B at 5.00 and 5.07 ppm were correlated to the anomeric carbon signals at 103.55 and 103.72 ppm, respectively. A and B were suggested to be →3)-α-l-Rha*p*-(1→ because of the downfield chemical shifts of the C-3 at 79.02 ppm as compared with that of parent α-l-rhamnopyranose residue. A and B with different chemical shifts probably corresponded to the same type of residue with different environments. The anomeric proton signals of C and D at 5.23 and 5.30 ppm, respectively, were correlated to the anomeric carbon signal at 102.41 ppm. C and D were assigned to →2)-α-l-Rha*p*-(1→, which was confirmed by the downfield chemical shift of the C-2 at 79.40 ppm. The anomeric proton signal of E at 5.39 ppm was related to the anomeric carbon signal at 102.37 ppm. E was proposed to be →2,3)-α-l-Rha*p*-(1→ on account of the downfield chemical shifts of the C-2 and C-3 at 79.9 and 79.0 ppm, respectively. 

Based on the analysis of the ^1^H–^1^H NOESY spectrum (Figure 1E), the repeating units of the monosaccharide sequences in the polysaccharide chain were established. The anomeric proton signal of A was correlated to the H-2 of C/D at 4.11 ppm and H-3 of E at 3.97 ppm, respectively, indicating the sequences →3)-α-l-Rha*p*-(1→2)-α-l-Rha*p*-(1→ and →3)-α-l-Rha*p*-(1→2,3)-α-l-Rha*p*-(1→. The anomeric proton signal of B was related to the H-3 of A at 3.89 ppm and H-3 of E at 4.11 ppm, respectively, indicating the presence of →3)-α-l-Rha*p*-(1→3)-α-l-Rha*p*-(1→ and →3)-α-l-Rha*p*-(1→2,3)-α-l-Rha*p*-(1→. The anomeric proton signal of C was related to the H-3 of A/B at 3.89 ppm, H-3 of E at 3.97 ppm and H-2 of D at 4.11 ppm, respectively, suggesting the presence of →2)-α-l-Rha*p*-(1→3)-α-l-Rha*p*-(1→, →2)-α-l-Rha*p*-(1→2,3)-α-l-Rha*p*-(1→ and →2)-α-l-Rha*p*-(1→2)-α-l-Rha*p*-(1→. In conclusion, the backbone of DSMS-1 was composed of →3)-α-l-Rha*p*-(1→, →2)-α-l-Rha*p*-(1→ and →2,3)-α-l-Rha*p*-(1→, and the branches were substituted partly at C-2 of →3)-α-l-Rha*p*-(1→ and/or C-3 of →2)-α-l-Rha*p*-(1→ residues. Structures of the main repeating disaccharides in DSMS-1 are shown in Figure 1F.

#### 2.2.3. NMR Spectroscopy Analysis of MS-1

**Figure 2:**
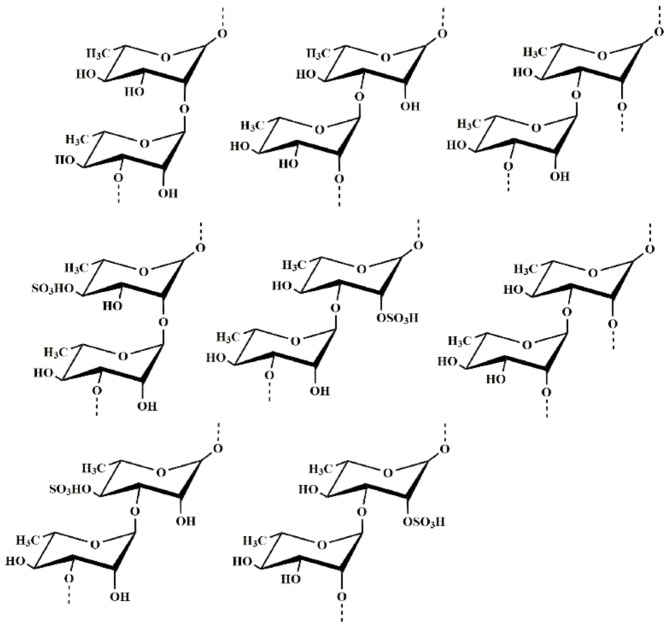
Structures of the main repeating disaccharides in MS-1.

In the ^1^H NMR spectrum of MS-1 (Figure S2A), there were six anomeric proton signals occurring at 5.07 (A), 5.23 (B), 5.25 (C), 5.39 (D), 5.45(E), and 5.50 ppm (F), with the relative integrals of 2.93:2.34:0.80:1.18:0.87:1.00. In the ^13^C NMR spectrum (Figure S2B) of MS-1, the anomeric carbon signals at 100.76~103.32 ppm were assigned to α-l-rhamnosyl units. Compared with the anomeric proton signals of DSMS-1, MS-1 had additional anomeric proton signals at 5.25, 5.45, and 5.50 ppm. By a comprehensive analysis of ^1^H NMR, ^13^C NMR, ^1^H–^1^H COSY (Figure S2C), and ^1^H–^13^C HSQC (Figure S2D) spectra, the anomeric proton signal of C at 5.25 ppm was related to the anomeric carbon signal at 101.85 ppm and C was ascribed to →2)-α-l-Rha*p*(4SO_4_)-(1→, with the downfield chemical shifts of the C-2 at 79.78 ppm and C-4 at 80.39 ppm. The anomeric proton signal of E at 5.45 ppm corresponded with the anomeric carbon signal at 100.40 ppm and E was assigned to →3)-α-l-Rha*p*(4SO_4_)-(1→, which was confirmed by the downfield chemical shifts of the C-3 at 79.35 ppm and C-4 at 81.99 ppm. The anomeric proton signal of F at 5.50 ppm was related to the anomeric carbon signal at 100.76 ppm and F was assigned to →3)-α-l-Rha*p*(2SO_4_)-(1→, due to the signals of the C-2 and C-3 shifted to 78.18 and 77.42 ppm, respectively. Based on the analysis of the ^1^H–^1^H NOESY spectrum (Figure S2E), the repeating units of the monosaccharide sequences in the polysaccharide chain were established. The anomeric proton signal of A was correlated to the H-3 of E at 4.00 ppm, H-3 of D at 4.09 ppm, H-3 of F at 4.11 ppm, H-2 of B at 4.28 ppm, and H-2 of C at 4.30 ppm, respectively, indicating the linkages →3)-α-l-Rha*p*-(1→3)-α-l-Rha*p*(4SO_4_)-(1→, →3)-α-l-Rha*p*-(1→2,3)-α-l-Rha*p*-(1→, →3)-α-l-Rha*p*-(1→3)-α-l-Rha*p*(2SO_4_)-(1→, →3)-α-l-Rha*p*-(1→2)-α-l-Rha*p*-(1→ and →3)-α-l-Rha*p*-(1→2)-α-l-Rha*p*(4SO_4_)-(1→. The anomeric proton signal of B was correlated with the H-3 of A at 3.96 ppm, H-3 of D at 4.09 ppm and H-3 of F at 4.11 ppm, respectively, indicating the sequences →2)-α-l-Rha*p*-(1→3)-α-l-Rha*p*-(1→, →2)-α-l-Rha*p*-(1→2,3)-α-L-Rha*p*-(1→ and →2)-α-l-Rha*p*-(1→3)-α-l-Rha*p*(2SO_4_)-(1→. The anomeric proton signal of D was correlated with the H-3 of A at 3.96 ppm and H-2 of B at 4.28 ppm, respectively, indicating the linkages →2,3)-α-l-Rha*p*-(1→3)-α-l-Rha*p*-(1→ and →2,3)-α-l-Rha*p*-(1→2)-α-l-Rha*p*-(1→. The anomeric proton signal of E was correlated with the H-3 of A at 3.96 ppm and H-3 of F at 4.11 ppm, respectively, suggesting the linkages →3)-α-l-Rha*p*(4SO_4_)-(1→3)-α-l-Rha*p*-(1→ and →3)-α-l-Rha*p*(4SO_4_)-(1→3)-α-l-Rha*p*(2SO_4_)-(1→. Besides the anomeric proton signal of F was correlated with the H-3 of A at 3.96 ppm, suggesting the linkage →3)-α-l-Rha*p*(2SO_4_)-(1→3)-α-l-Rha*p*-(1→. Structures of the main repeating disaccharides in MS-1 were shown in Figure 2. The assignment of the main signals of the six sugar residues could be completed (Table 2). 

#### 2.2.4. Hydrophilic Interaction Liquid Chromatography-Fourier-Transform Mass Spectrometry Analysis of MS-1-Derived Oligosaccharides

In order to get more detailed structural information, MS-1 was partially hydrolyzed by mild acid. Two hydrolysis products, the supernatant (MS-1-O) and precipitate (MS-1-P) products were obtained. Compared with the high performance liquid chromatography (HPLC) result of MS-1, the monosaccharide composition of MS-1 had slightly change. The molar percent ratios of rhamnose, glucuronic acid, glucose and xylose in MS-1-P were ~94.44:2.51:1.72:1.33, while MS-1-O contained a large amount of rhamnose, xylose and glucose with a minor amount of glucuronic acid (Figure S3A). The results indicated that the rhamnose, xylose, glucose and glucuronic acid could be released from the chain of MS-1 by mild acid hydrolysis. Hence, it could be deduced that the side chain of MS-1 consisted of glucose, xylose, rhamnose, and glucuronic acid. 

MS-1-O was followed by hydrophilic interaction liquid chromatography-Fourier-transform mass spectrometry (HILIC-FTMS) analysis. The total ion chromatogram of MS-1-O is shown in Figure S3B. The raw data was deconvoluted using Decon tools, and then was processed by GlycResoft to get matching structures and relative quantitative information [21,22]. Relative quantitative results of the oligosaccharides were shown in Figure S3C. A total of 22 chain compositions with size equal or smaller than degree of polymerization (dp) 6 were detected in MS-1-O, in which G_2_ (24.55%), G_3_ (14.99%), R_2_S (8.23%), X_2_ + G (19.57%), R_3_S (5.48%), X_2_ + G + R(S) (8.02%), and R_2_S + GA/R_2_ + GA(S) (4.13%) were the main components. Minor amounts of R_2_S_2_, X + R, R_4_S_2_, RS + GA/R + GA(S), R_5_S_2_, and R + GA were also detected. The content of other oligosaccharides was below 1%. R, G, GA, X, and S represented rhamnose, glucose, glucuronic acid, xylose and sulfate ester, respectively. Here, the detailed sequences of the oligosaccharides containing glucuronic acid were further investigated. 

The negative ion MS^2^ spectrum of R + GA at *m*/*z* 339 was given in Figure S4A. The major fragment at *m*/*z* 321 was derived from the dehydration of the ion at *m*/*z* 339, and the less intensive fragment at *m*/*z* 193 arose from glyosidic bond cleavages of C_1_. No ^2,5^A-type cross-ring cleavage was detected, which is a unique cleavage from reducing end GlcA residue [23], indicating the GlcA was at the nonreducing end. The presence of C_1_ suggested the 1→3 linkage between GlcA and Rha, because the fragmentation of the 3-*O*-sulfated monosaccharide led to only one high abundance daughter ion at *m*/*z* 97 [24]. The minor ion at *m*/*z* 235 which was originated from ^0,2^A indicated that minor amount of the 1→2 linkage was also existed between GlcA and Rha. So, the sequence of R + GA might be GlcA(1→3)Rha and/or GlcA(1→2)Rha, and the former was the main component.

The negative ion MS^2^ spectrum of RS + GA/R + GA(S) at *m*/*z* 419 was listed in Figure S4B, the most abundant ion at *m*/*z* 255 arose from glyosidic bond cleavage of B_1_, indicating the presence of sulfated glucuronic acid and the sulfate ester could be at the C-2 of the GlcA according to NMR analysis. The less-intensive ion at *m*/*z* 243 arose from glyosidic bond cleavage of Z_1_, indicating the 1→3 linkage between GlcA and Rha. The minor ion at *m*/*z* 183 arose from cross-ring cleavage ^0,2^A_2_, indicating the 1→2 linkage between GlcA and Rha. So, the sequence of RS + GA/R + GA(S) might be GlcA(2SO_4_)(1→3)Rha, GlcA(2SO_4_)(1→2)Rha, GlcA(1→3)Rha(4SO_4_), GlcA(1→3)Rha(2SO_4_), and/or GlcA(1→2)Rha(4SO_4_).

The negative ion MS^2^ spectrum of R_2_S + GA/R_2_ + GA(S) at *m*/*z* 565 was listed in Figure S4C. The ions at *m*/*z* 255, 193, 321, and 339 arose from the glyosidic bond cleavages of B_1_, B_2_, C_1_, and C_2_, respectively. The ion at *m*/*z* 255 indicated the presence of the sulfated glucuronic acid. The ions at 225, 243, and 389 arose from the glyosidic bond cleavages of Y_1_, Z_1_, and Y_2_, respectively. The ions at *m*/*z* 225 and 243 indicated the presence of the sulfate ester at the reducing end of rhamnose. The main structures of R_2_S + GA/R_2_ + GA(S) were GlcA(2SO_4_)(1→3)Rha(1→3)Rha GlcA(2SO_4_)(1→3)Rha(1→2)Rha, GlcA(2SO_4_)(1→2)Rha(1→3)Rha, GlcA(2SO_4_)(1→2)Rha(1→2)Rha, GlcA (1→3)Rha(1→3)Rha(2SO_4_), GlcA (1→3)Rha(1→3)Rha(4SO_4_), GlcA(2SO_4_)(1→2)Rha(1→3)Rha(2SO_4_) and/or GlcA(2SO_4_)(1→2)Rha(1→3)Rha(2SO_4_). 

The above results demonstrated that the backbone of MS-1 mainly consisted of →3)-α-l-Rha*p*-(1→ and →2)-α-l-Rha*p*-(1→ residues, partially sulfate groups were at C-4/C-2 of (1→3)-Rha*p* and C-4 of (1→2)-Rha*p* residues. The branching was composed of sulfated or unsulfated terminal β-d-GlcA*p*, →3)-α-l-Rha*p*-(1→ and →2)-α-l-Rha*p*-(1→, as well as →4)-β-d-Xyl*p*-(1→, →4/→6)-d-Glc*p*-(1→ residues. The branching substituted at C-2 of →3)-α-l-Rha*p*-(1→ and C-3 of →2)-α-l-Rha*p*-(1→ residues. MS-1 had sophisticated structural characteristics differing from those of the sulfated polysaccharides previously obtained from Monostroma species [15,16,25,26,27]. The structural changes may be due to the species, ecophysiological growth conditions, and harvest seasons [28]. The microheterogeneities with sugar residues and/or sulfation patterns of sulfated polysaccharides could have originated from sequential enzymatic modification in the biosynthesis of these molecules [29]. The present result suggested that the marine green algae could be a potential source of sulfated polysaccharides with unique structures, and be worth being further investigated.

### 2.3. Anticoagulant Activity In Vitro and In Vivo of MS-1 and Its Platelet Aggregation

Activated partial thromboplastin time (APTT), thrombin time (TT) and prothrombin time (PT) are widely used for estimating the anticoagulant activity. The prolongation of APTT indicates inhibition of the intrinsic and/or common pathway. TT reflects the blood coagulation status that transforming fibrinogen into fibrin [30]. PT is the extrinsic pathway-dependent clotting time. The anticoagulant activity of MS-1 in vitro was evaluated by APTT, TT and PT assays using heparin as a reference. 

As shown in Table 3, the anticoagulant activity of MS-1 was concentration-dependent. MS-1 effectively prolonged the APTT, and the clotting time was more than 200 s at 25 µg/mL. In addition, the TT activity by MS-1 slowly increased, and the clotting time was more than 120 s at 50 µg/mL. It was observed that the APTT and TT activities of MS-1 were different from that of heparin. Heparin quickly increased the APTT and TT times; the clotting time was more than 200 s at 10 µg/mL for APTT and 120 s at 10 µg/mL for TT. Lack of prolongation effect of MS-1 on the PT was also observed with increasing concentration of MS-1. The results showed that MS-1 inhibited both the intrinsic and/or common pathways of coagulation and thrombin activity or conversion of fibrinogen to fibrin. 

**Figure 3:**
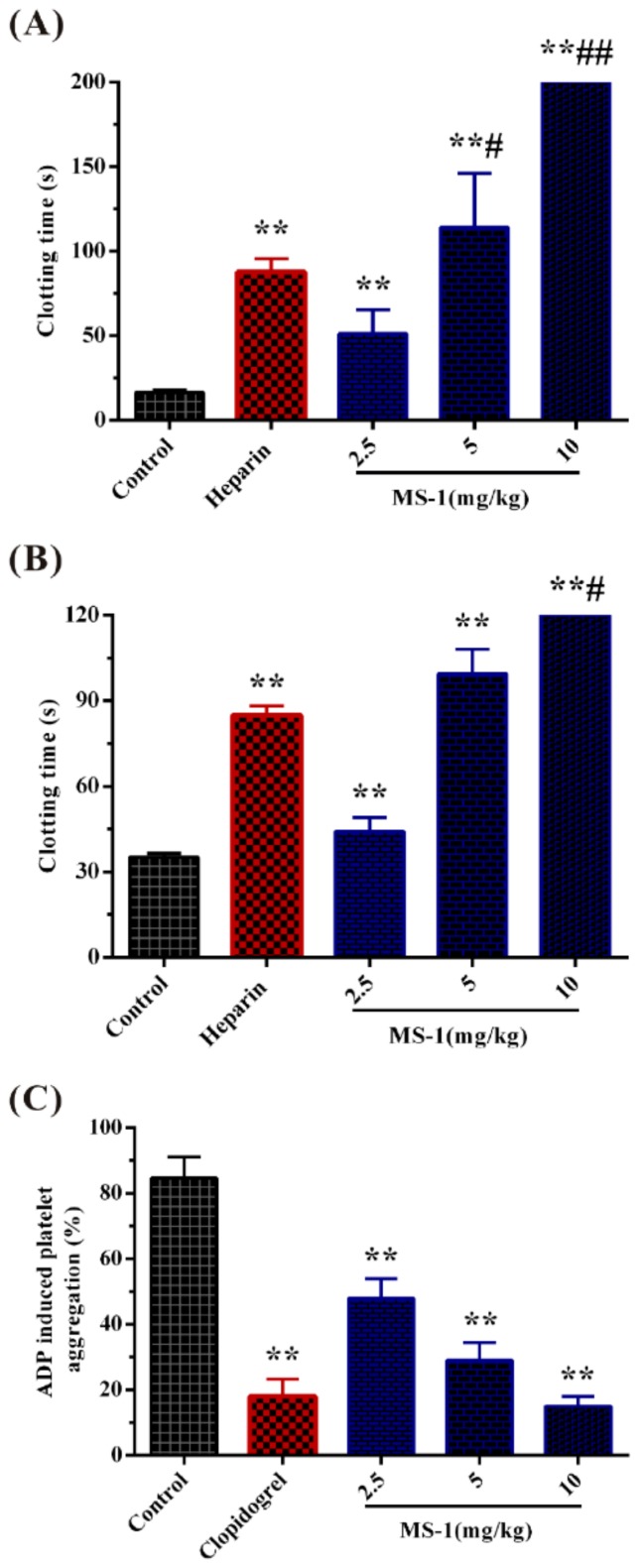
Anticoagulant activity and in vivo and platelet aggregation of MS-1. (**A**) APTT, (**B**) TT, and (**C**) platelet aggregation, clopidogrel. * *p* < 0.05, ** *p* < 0.01 versus control, ^#^ *p* < 0.05, ^##^ *p* < 0.01 versus the heparin or clopidogrel group.

Anticoagulant activity of MS-1 in vivo was further evaluated by assays of APTT, PT, and TT. No rats were found moribund and bleeding after intravenous injection of heparin and MS-1. Furthermore, the clotting times had been prolonged after injection of MS-1 and heparin, indicating that heparin and MS-1 were absorbed. As shown in Figure 3A,B, MS-1 indicated significant prolongation effects on the APTT and TT at 2.5 mg/kg, and no prolongation effect on the PT was found (data not shown). Furthermore, the APTT activity of MS-1 had exceeded that of heparin at 5 mg/kg. Thus, MS-1 exhibited strong anticoagulant activity in vivo.

MS-1 had a higher anticoagulant activity in vitro than some sulfated polysaccharides from Monostroma species [17,18,25,26,27]. It is interesting to note that the APTT activity of MS-1 was weaker than that of the sulfated polysaccharide WF3 from *M. nitidum* [31], though both of MS-1 and WF3 mainly consisted of →2)-α-l-Rha*p*-(1→ and →3)-α-l-Rha*p*-(1→ residues. The sulfate ester of MS-1 was substituted mainly at C-2/C4 of →3)-α-l-Rha*p*-(1→ and C4 of →2)-α-l-Rha*p*-(1→ residues, while the sulfate ester of WF3 was at C-2 of →3)-α-l-Rha*p*-(1→ residue. In addition, no anticoagulant activity was found in DSMS-1 (data not shown), indicating that the sulfate ester content played a major role in the anticoagulant activity of sulfated polysaccharide. Mao et al. [31] also reported that the sulfate ester content markedly modified the anticoagulant activity of sulfated rhamnan. The results demonstrated that the sulfated polysaccharides from Monostroma species exhibited anticoagulant activity not merely as a function of charge density. The structural basis of anticoagulant activity depends on the sites of sulfation, and also on the distribution and/or the proportion of sulfation pattern.

Platelet aggregation plays a central role in the extension artery thrombosis and adenosine diphosphate (ADP) induced primary and secondary aggregation [32]. This aggregation involves purinergic receptors that are targets for generation of new antiplatelet agents, such as clopidogrel. The antiplatelet aggregation activity of MS-1 was evaluated via a conventional turbidimetric assay using clopidogrel as a positive control. As shown in Figure 3C, MS-1 could significantly inhibit platelet aggregation, and the inhibitory effect was concentration-dependent. Furthermore, the inhibitory effect on the platelet aggregation of MS-1 at 10 mg/kg was higher than that of clopidogrel. The concentration of clopidogrel used in the experiment referred to the clinical dosage in human according to the specification of the clopidogrel. The data suggested that MS-1 had a high antiplatelet aggregation activity. So far, little work in the literature is related to the antiplatelet aggregation property of sulfated polysaccharides from marine green algae. The effect of platelet aggregation could relate to the structural variety of sulfated polysaccharides [33]. Further work is required to investigate the platelet aggregation of the sulfated polysaccharides from Monostroma species.

### 2.4. Effects of MS-1 on Thrombin and Factor Xa Activities Mediated by AT-III or HC-II

**Figure 4:**
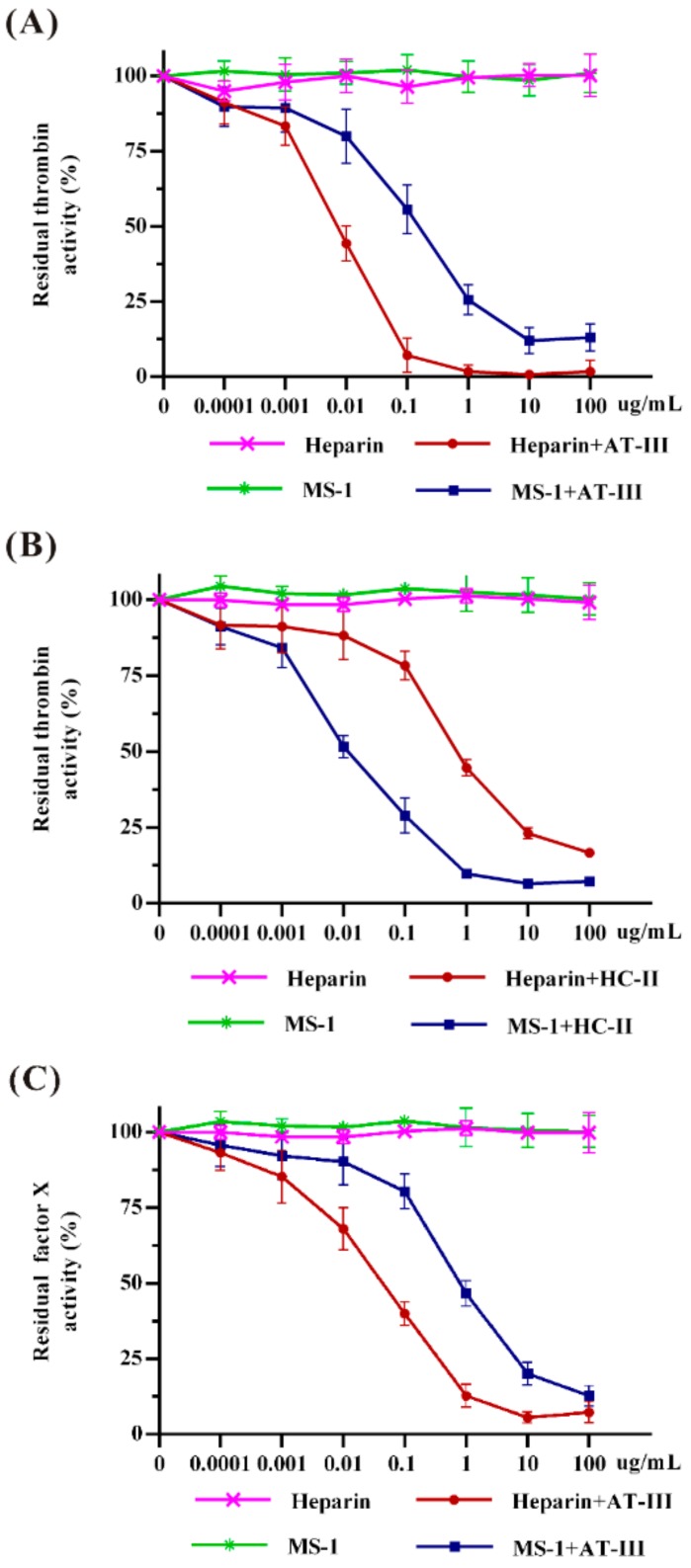
Effects of MS-1 on thrombin and factor Xa activities. (**A**) The activity of thrombin in the absence and presence of AT-III; (**B**) the activity of thrombin in the absence and presence of HC-II; and (**C**) the activity of factor Xa in the absence and presence of HC-II.

In order to elucidate the inhibitory mechanism of MS-1, a specific analysis for coagulant factors Xa and thrombin inhibitions mediated by antithrombin-III (AT-III) and heparin cofactor-II (HC-II) were performed using chromogenic assays in vitro. As shown in Figure 4, both heparin and MS-1 were observed to poorly inhibit thrombin and factor Xa in the absence of HC-II or AT-III. However, thrombin inhibition was pronounced after combined treatment with AT-III or HC-II in a dose-dependent manner. It was noted that the effect of MS-1 on thrombin inhibition mediated by HC-II was stronger than that of heparin. However, a higher concentration of MS-1 was required to obtain the same thrombin inhibition as heparin mediated by AT-III. In addition, MS-1 had a strong inhibitory effect on the activity of factor Xa in the presence of HC-II, but it had no inhibitory effect on factor Xa in the presence of AT-III (data not shown). 

Normally, AT-III inhibits all intrinsic pathway coagulation enzymes, and HC-II is a serine protease inhibitor and selectively inhibits thrombin. Inherited deficiency of AT-III individuals follows hypercoagulable disorders [34]. The results suggested that MS-1 did not have direct inhibition of thrombin and factor Xa. MS-1 was a potent thrombin inhibitor mediated by HC-II. It also hastened thrombin and factor Xa inhibitions mediated by AT-III, but MS-1 is less effective than heparin. The differences may be attributed to their structural features variation. MS-1 and heparin bind to different sites on AT-III. The conformational activation of AT-III and the consequent formation of a covalent complex with thrombin or factor Xa appear to be less important for anticoagulant activity of MS-1 than for heparin. Hayakawa reported that the effect of sulfated polysaccharide from Monostroma species on thrombin inhibition mediated by HC-II was stronger than that of heparin [35]. It is observed that the effect of MS-1 on thrombin and factor Xa inhibitions mediated by AT-III or HC-II had some differences from those of the sulfated polysaccharides from Monostroma species [27,31]. The differences between thrombin and factor Xa inhibitions may be attributed to the variation of their molecular size, charge density, sulfation position and the linkage pattern of rhamnose residues [36]. Structural requirements for the interaction of sulfated polysaccharide with coagulation inhibitors and their target proteases were stereospecific and not merely a consequence of charge density [37]. An in-depth study in the relationship between the structure and mechanism of anticoagulant activity of Monostroma sulfated polysaccharides is required.

### 2.5. Fibrinolytic and Thrombolytic Activities of MS-1

**Figure 5:**
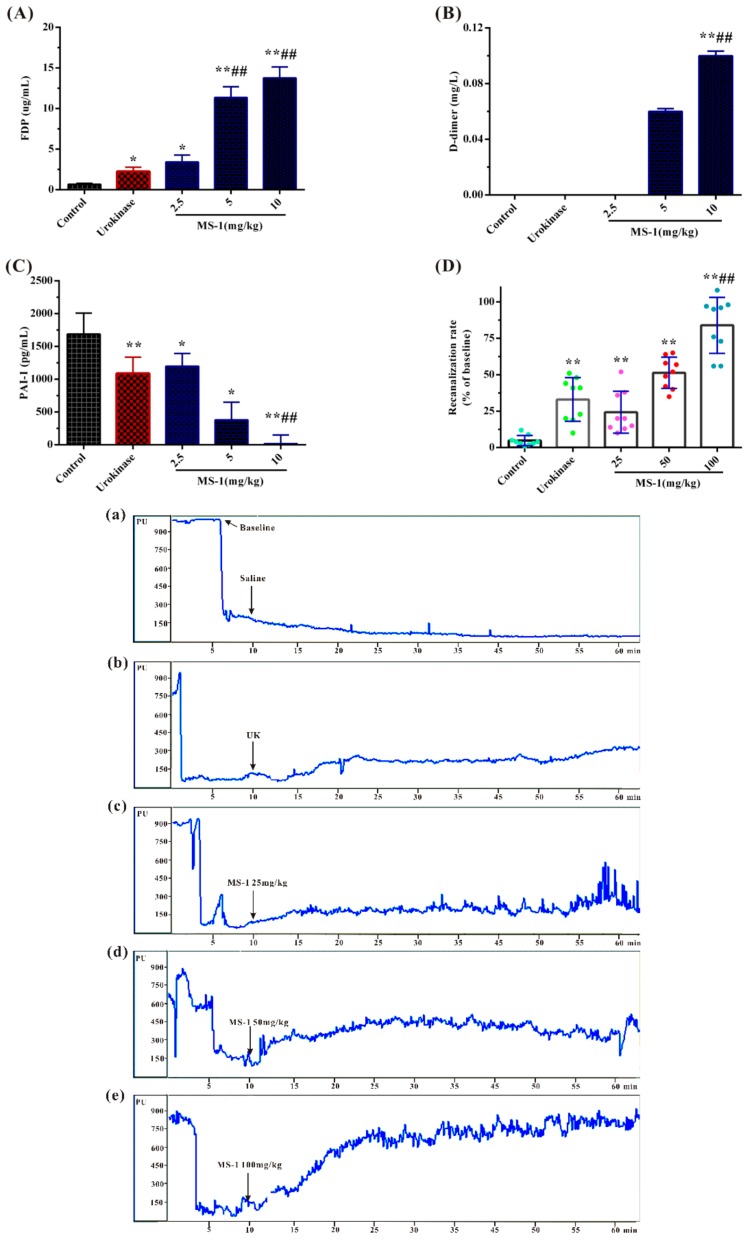
Results of fibrinolytic and thrombolytic activities in vivo of MS-1. (**A**) FDP; (**B**) d-dimer, the d-dimer value of control and urokinase was below 0.05 mg/L; (**C**) PAI-1; and (**D**) recanalization rate, time–carotid artery blood flow curves of mice injected saline (a: saline; b: urokinase; c: 25 mg/kg MS-1; d: 50 mg/kg MS-1; d: 100 mg/kg MS-1). Significance: * *p* < 0.05, ** *p* < 0.01 versus the control group; ^#^ *p* < 0.05, ^##^ *p* < 0.01 versus the urokinase group.

The fibrinolytic activity of MS-1 was evaluated by fibrin degradation products (FDP) plasminogen activator inhibitor-1 (PAI-1) and d-dimer levels using rat plasma; urokinase was used as a reference. FDP is the degradation product of fibrous protein and is involved in many vascular diseases [38]. d-dimer is a specific protein fiber degradation product of cross-link fibrin by the hydrolysis of fibrinolytic enzyme [39,40]. Fibrinolytic inhibitor PAI-1 is a potent inhibitor of the fibrinolytic agent tissue-type plasminogen activator and urokinase-type plasminogen activator (uPA) and determines resistance to artery thrombolysis [41]. PAI-1 has heightened fibrinolytic capacity and exhibits endogenous protection against thrombosis, which could bind the ternary complex PAI-1–uPA–uPA receptor (uPAR) thereby exerting antifibrinolytic activity effect [42,43]. As shown in Figure 5A,B, the levels of FDP and D-dimer were obviously increased by MS-1. The increasing effects of MS-1 at 5 and 10 mg/kg on the levels of FDP and d-dimer were significantly higher than that of urokinase in the concentration used in the experiment. Compared with the control group, the level of PAI-1 was effectively decreased by MS-1 (Figure 5C). Moreover, the decreasing effects of MS-1 at 5 and 10 mg/kg on PAI-1 were significantly higher than that of urokinase. The results demonstrated that MS-1 had a high fibrinolytic activity in vitro. MS-1 appeared to depress the activity of PAI-1, release uPA–uPAR complex who activated proenzyme plasminogen to biologically active plasmin, promote fibrin degradation and result in increasing of FDP and D-dimer levels. 

Further, the effect of MS-1 on carotid artery thrombosis in vivo induced by FeCl_3_ was investigated by monitoring blood flow using urokinase as a reference. As shown in Figure 5Da, after 3 min of FeCl_3_ stimulation, the blood flow of common carotid artery was rapidly reduced to occlusion in all experimental groups, and no increase in the control group occurred after saline injection. From Figure 5Db–e, a reversion was found in positive control and MS-1 groups. Effective recanalization rate was found in MS-1 groups, and it was in a dose-dependent manner. Obviously, the blood flow treated with 25 mg/kg of MS-1 increased to 24.22% of the baseline, the blood flow treated with 50 and 100 mg/kg of MS-1 groups increased to 51.33% and 83.89%, respectively, which exceeded that of urokinase group. The results indicated that the occluded carotid artery could be recanalized after intravenous injection MS-1, and complete recanalization could be achieved at a high concentration of MS-1. Carotid artery thrombus occurs in vessels with severe atherosclerotic disease and may embolize to cause transient ischemic attacks and cerebral infarctions, associating with severe iron deficiency anemia and thrombocytosis [1]. The basis of therapy is the intravenous administration of an exogenous plasminogen activator [44]. An in-depth work is required to investigate the thrombolytic activity of Monostroma sulfated polysaccharides.

### 2.6. Antithrombotic Activities In Vitro and In Vivo of MS-1

**Figure 6:**
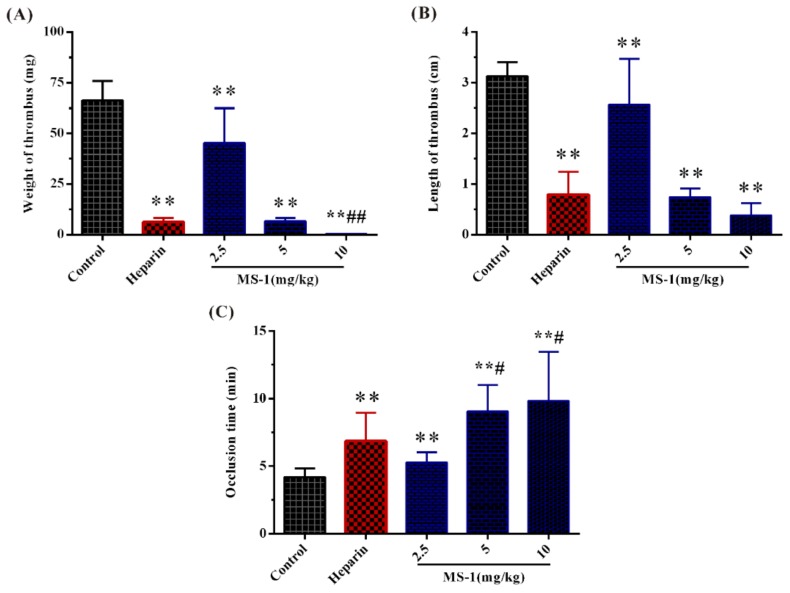
Antithrombotic effect of MS-1. (**A**) Thrombus dry weight, (**B**) thrombus wet length, and (**C**) occlusion time. Significance: * *p* < 0.05, ** *p* < 0.01 versus the control group; ^#^ *p* < 0.05, ^##^ *p* < 0.01 versus the heparin group.

Antithrombotic activities of MS-1 in vitro and in vivo were investigated by assays of the weight and length of thrombus and the carotid artery occlusion time by electrical induced carotid artery thrombosis model using heparin as a reference. As shown in Figure 6A,B, the wet weights and lengths of the thrombus for MS-1 groups were far below those of the control, MS-1 could significantly inhibit the thrombus formation in a dose-dependent manner. Moreover, it was observed that the weights of thrombus of MS-1 at 5 and 10 mg/mL were lighter than that of heparin in the concentration used in the experiment. Similar results were also found in the wet lengths of thrombus. The results suggested that MS-1 had a high antithrombotic activity in vitro. 

Antithrombotic activity of MS-1 in vivo was further evaluated. The carotid artery thrombosis induced by electrical consisted of large amounts of platelets and red cells which involved in a fibrin network [36]. As shown in Figure 6C, the occlusion times of the thrombus for MS-1 groups were significantly increased. The effects of MS-1 at 5 and 10 mg/kg were stronger than that of heparin. The notable potency of MS-1 observed in the carotid artery thrombosis model is related to its potent inhibitory effect in the coagulation and platelet aggregation. It is noted that some sulfated polysaccharides exhibit antithrombotic activity and are heparinoid-active [45,46,47]. However, little work in the literature is related to the antithrombotic activity of sulfated polysaccharides from Monostroma species. Further work is required to investigate the antithrombotic effect of Monostroma sulfated polysaccharides.

Marine algae produce a large amount of sulfated polysaccharides with various biological activities. In view of the increasing incidence of thrombotic diseases, effective functional foods or drugs are urgently desired. Until now, the research on anticoagulant and antithrombotic activities of sulfated polysaccharides from marine green algae is still less comprehensive than those of sulfated polysaccharides from brown and red algae. The present results revealed that MS-1 from marine green alga *M. nitidum* possessed strong anticoagulant and antithrombotic activities in vitro and in vivo, and could be a promising drug or functional foods for prevention and therapy of thromboembolic disease. An in-depth investigation on MS-1 is in progress.

## 3. Materials and Methods

### 3.1. Materials

*M. nitidum* was collected from the coast of Yantai, China on May 2016. The raw material was thoroughly washed with tap water, air-dried, milled using a blender, and then stored at room temperature in a dry environment. Q Sepharose Fast Flow and Sephacryl S-400/HR were from GE Healthcare Life Sciences (Piscataway, NJ, USA). Dialysis membranes (flat width 44 mm, molecular weight cut-off 3500; flat width 31 mm, molecular weight cut-off 1000) were from Lvniao (Yantai, China). Pullulan standards (Mw: 9.6, 21.1, 47.1, 107, 200, 344 and 708 kDa) were from Showa Denko K.K. (Tokyo, Japan). l-rhamnose, l-arabinose, d-xylose, d-fucose, d-mannose, d-galactose, d-glucose, d-glucuronic acid, d-galacturonic acid, and d-glucosamine were from Sigma (St. Louis, MO, USA). APTT, TT, and PT kits were from MD Pacific (Tianjin, China). Heparin (196 U/mg) was obtained from Sigma (St. Louis, MO, USA). Clopidogrel was from Sanofi (Hangzhou, China). Urokinase was obtained from Ndpharm (Nanjing, China). d-Dimer kit was from Siemens Healthcare Diagnostics Products (Marburg, Germany). Factor Xa and thrombin were from Boatman Biotech CO., LTD. (Shanghai, China). AT-III was from Chromogenix (Milan, Italy). HC-II was from Hyphen Biomed (Neuville, France). S-2238 and S-2765 were from Asnail (Beijing, China). FDP kit was from BIOLINKS CO., LTD. (Tokyo, Japan). PAI-1 kit was from Cloud-Clone Corp (Wuhan, China).

### 3.2. Animals

Male Sprague-Dawley (SD) rats (180–220 g) and Kunming (KM) mice (18–20 g) were housed at 23 ± 2 °C under a 12 h light/dark cycle with free access to food and water. All animal experiments were approved by the Institutional Animal Care and Use Committee of the Ocean University of China (OUC-YY-201801001).

### 3.3. Isolation and Purification of the Sulfated Polysaccharide

The alga powder (100 g) was dipped into 40 vols of distilled water and extracted at 100 °C for 4 h, and then centrifuged at 3600× *g* for 15 min, concentrated, and dialyzed in a cellulose membrane against distilled water. The retained fraction was recovered, concentrated by rotary evaporation, precipitated by adding four volumes of 95% ethanol (v/v) and dried at 40 °C to obtain a crude polysaccharide. The crude polysaccharide was fractionated on a Q Sepharose Fast Flow column (30 cm × 3.5 cm, GE Healthcare Life Sciences, USA), and eluted with a stepwise gradient of 0, 0.5, 1.0, 1.5, 2.0, 2.5, 3.0, and 3.5 mol/L NaCl at a flow rate of 1 mL/min. Eluate was collected by autofraction collector (8 mL/tube, Bio-Rad, Hercules, CA, USA). Total sugar content of the eluate was determined by the phenol–sulfuric acid method [48]. The subfraction eluted with 0.5 mol/L NaCl was pooled and dialyzed. Then, the fraction was further purified on a Sephacryl S-400/HR column (100 cm × 2.5 cm; GE Healthcare Life Sciences, USA), eluted with 0.2 mol/L NH_4_HCO_3_ at a flow rate of 0.3 mL/min. Eluate was collected by autofraction collector (6 mL/tube). The major fraction of MS-1 was pooled and freeze-dried (1.34 g).

### 3.4. General Technique of Structural Characteristics

The homogeneity and molecular weight were determined by HPGPC on a Shodex OHpak SB-804 HQ column (7.8 mm × 300 mm, Tokyo, Japan) and elution with 0.2 mol/L Na_2_SO_4_ at a flow rate of 0.5 mL/min. The molecular weight was estimated by reference to a calibration curve made by pullulan standards [49]. Total sugar content was assayed by the phenol–sulfuric acid method using rhamnose as the standard. Protein content was determined as described by Bicinchoninic acid protein assay kit (Shanghai, China). Sulfate ester content was measured according to the method of Therho and Hartiala [50]. Uronic acid content was determined by the carbazole–sulfuric acid method using glucuronic acid as standard [51]. Monosaccharide compositions were measured by reversed phase HPLC after precolumn derivatization [25]. Calculation of the molar ratio of the monosaccharide was carried out on the basis of the ratio of peak areas of monosaccharide and correspondent monosaccharide standard. Sugar configuration was determined according to the method of Tanaka et al. [52]. Identification of sugar configuration was completed by comparison with retention time of the derivatives of reference sugars.

Desulfation of the sulfated polysaccharide was performed according to the method of Falshaw and Furneaux [53]. Desulfurization product of MS-1 was named DSMS-1. The effectiveness of desulfation procedure was confirmed by determination of residual sulfate in the polysaccharide. Methylation analysis was performed according to the method of Sims et al. [54] with some modification. The completion of methylation was confirmed by IR spectroscopy as the disappearance of OH bands. The products were analyzed by gas chromatography–mass spectrometry (GC–MS) on a TRACE 1300 instrument (Thermo Fisher, Waltham, MA, USA) using a DB 225 fused silica capillary column (0.25 mm × 30 m, Agilent Technologies Co. Ltd., Palo Alto, CA, USA). Identification of partially methylated alditol acetates was carried out on the basis of retention time and mass fragmentation patterns.

### 3.5. Spectroscopy Analysis

FTIR spectrum of the polysaccharide was measured on a Nicolet Nexus 470 spectrometer (Thermo Fisher Scientific, Waltham, MA). The polysaccharide was mixed with KBr powder, ground and pressed into a 1 mm pellet for FTIR measurements in the frequency range of 4000–400 cm^−1^ [55]. ^1^H and ^13^C NMR spectra were performed at 23 °C on an Agilent DD2 500 M NMR spectrometer (Agilent Technologies CO., LTD., USA). Approximately 70 mg of polysaccharide was dissolved in 1 mL of D_2_O (99.97%) followed by freeze-drying for three times, and the final sample was dissolved in 0.5 mL of 99.97% D_2_O. ^1^H–^1^H COSY, ^1^H–^13^C HSQC, and ^1^H–^1^H NOESY experiments were also carried out. Chemical shifts are expressed in ppm using acetone as an internal standard at 2.23 ppm for ^1^H and 31.07 ppm for ^13^C.

### 3.6. Profiling of MS-1-Derived Oligosaccharides by HILIC-FTMS 

The acid hydrolysis of MS-1 was performed according to the method of [56] with some modifications. MS-1 (10 mg/mL) was hydrolyzed with 0.2 mol/L TFA at 60 °C for 1 h. The hydrolysis product was neutralized with 0.2 mol/L NaOH. After concentrated, four-fold volume of 95% (v/v) ethanol was added. The resulting supernatant and precipitate were recovered by centrifugation (6000× *g*, 10 min), designated as MS-1-P and MS-1-O, respectively. The precipitate MS-1-P was washed with ethanol and vacuum-dried. The supernatant MS-1-O was concentrated, freeze-dried and redissolved in 50 μL of 50% acetonitrile for HILIC-FTMS analysis.

HILIC-FTMS analysis was performed on an Agilent 1290 LC UPLC system (Wilmington, DE, USA) equipped with LTQ ORBITRAP XL mass spectrometer (Waters, Milford, MA, UK). The oligosaccharides were separated by a Luna HILIC column (2.0 mm × 50 mm, Phenomenex, Torrance, CA, USA) at 25 °C. The mobile phase was a mixture of 5 mmol/L 98% of NH_4_OAc acetonitrile (solvent A) and 5 mmol/L NH_4_OAc/H_2_O (solvent B) at a flow rate of 150 μL/min. The gradient was programmed as 98% A in the beginning, and linearly changed to 60% A at 10 min and 30 min. The analysis was performed in the negative ion mode using a capillary temperature of 275 °C. The spray voltage was 4.2 KV and nitrogen dry gas flowed at 40 L/min. Data analysis were performed using GlycReSoft 1.0 software [57,58].

### 3.7. Assessment of Anticoagulant Activity and Platelet Aggregation 

#### 3.7.1. Anticoagulant Activity In Vitro

APTT, TT, and PT assays were carried out according to the manufacturer’s instructions. For the APTT clotting assay, 95 μL of citrated sheep plasma was mixed with 5 μL of the sample solution (0.9% NaCl) at various concentrations and incubated at 37 °C for 60 s. Then, 100 μL of prewarmed APTT reagent was added to 100 μL of mixture plasma and allowed to incubate at 37 °C for 180 s. Thereafter, prewarmed 0.025 mol/L CaCl_2_ (100 μL) was added, and clotting time was recorded in a SL318 coagulometer (Senlan Medical Science and Trading CO., LTD., Jinan, China). For the PT clotting assay, 95 μL of citrated sheep plasma was mixed with 5 μL of the sample solutions (0.9% NaCl) at various concentrations and incubated at 37 °C for 180 s. Then, 200 μL of prewarmed PT assay reagent was added and clotting time was recorded. For the TT clotting assay, 95 μL of citrated sheep plasma was mixed with 5 μL of the sample solutions (0.9% NaCl) at various concentrations and incubated at 37 °C for 60 s. Then, 200 μL of prewarmed TT assay reagent was added and clotting time was recorded. Heparin was used for the comparison of anticoagulant activity of MS-1. Saline solution (0.9% NaCl) was used as control.

#### 3.7.2. Anticoagulant Activity In Vivo and Platelet Aggregation

Male SD rats were randomly divided into five groups, each group included 12 rats. The experimental rats were anaesthetized with 15% urethane, and then injected with MS-1 (2.5, 5, and 10 mg/kg), heparin (0.5 mg/kg), or saline solution. After 30 min, blood was drawn from the abdominal aorta and collected into blood collection tube with 3.8% sodium citrate (citrate/blood = 1/9, v/v). APTT, PT, and TT activity in vivo were determined by above mentioned methods.

The platelet aggregation was determined by the turbidimetric method using an aggregometer according to Xin et al. [59]. Platelet-rich plasma (PRP) was prepared by low speed centrifugation at 1000 × *g* for 15 min at room temperature. After the PRP was collected, the residual blood was further centrifuged at 3000 × *g* for 10 min at room temperature to obtain platelet-poor plasma (PPP). The PRP was regulated by the PPP to the concentration of platelets of 5 × 10^8^/mL. For the aggregation experiment, 290 μL of PRP were prewarmed at 37 °C for 5 min. Subsequently, ADP (5 μmol/L) was added to initiate platelet aggregation with stirring. The changes in light transmission were recorded for 5 min. Clopidogrel (75 mg/kg) was used as positive control and saline was used as a control.

### 3.8. Effects of MS-1 on the Thrombin and Factor Xa Activities Mediated by AT-III or HC-II

The assays were performed according to the method of Colliec et al. [60]. Human thrombin or coagulation factor Xa and inhibitors (HC-II or AT-III) were incubated with or without MS-1 in 180 μL 0.02 mol/L trisaminomethane (Tris)-HCl, 0.15 mol/L NaCl and 1.0 mol/L polyethylene glycol (PEG) at 37 °C. After 120 s incubation, 20 μL of Tris–HCl buffer containing 1.5 mmol/L chromogenic substrate S-2238 for thrombin or S-2765 for coagulation factor Xa was added, and the residual thrombin or coagulation factor Xa activities were determined by measuring the change in the absorbance at 405 nm. The change rate of absorbance was proportional to the thrombin or coagulation factor Xa activity remaining in the incubation. Heparin was used as a positive control and saline was used as a control.

### 3.9. Fibrinolytic and Thrombolytic Activities

#### 3.9.1. Fibrinolytic Activity 

Male SD rats were randomly divided into five experimental groups (12 rats/group). The experimental rats were anaesthetized, and then injected with MS-1 (2.5, 5, and 10 mg/kg), urokinase (20,000 U/kg) or saline solution. After 30 min to allow for circulation, the rats were secured in the supine position, and the blood was drawn from the abdominal aorta and collected into a tube with 3.8% sodium citrate (citrate/blood = 1:9, v/v) as a plasma anticoagulant. The level of PAI-1 was determined with ELISA kit through enzymatic marker (Bio Tek, Burlington, VT, USA). FDP and D-dimer assays were determined with ELISA kits through a CS-5100 automated blood coagulation analyzer (Sysmex Corporation, Kobe, Japan).

#### 3.9.2. FeCl_3_-Induced Carotid Artery Thrombosis

The model of FeCl_3_-induced carotid artery thrombosis was established on the basis of procedures described previously with modifications through an ultrasonic Doppler flow probe [61]. Male KM mice were anesthetized with 15% urethane. The carotid arteries were lightly exposed under a surgical microscope. An ultrasonic Doppler flow probe was placed on the surface of the carotid artery and blood flow was recorded using a PeriFlux System 5000 instrument (Stockholm, Sweden). Vascular injuries were generated by applying a filter paper (1 cm length and 3 mm width) saturated with 7.5% FeCl_3_ on top of the left carotid artery for 3 min. Then the carotid arteries were washed with normal saline and blood flow was complete occlusion after 10 min. Then, mice were injected with MS-1 (25, 50, and 100 mg/kg), urokinase (100,000 U/kg) or saline solution and the blood flow was recorded.

### 3.10. Assessment of Antithrombotic Activity 

#### 3.10.1. Experimental Thrombosis In Vitro

Male SD rats were randomly divided into five experimental groups (12 rats/group). The experimental rats were anaesthetized, and then injected with MS-1 (2.5, 5, and 10 mg/kg), heparin (0.5 mg/kg) or saline solution. After 30 min to allow for circulation, the rats were secured in the supine position, and the blood was drawn from the abdominal aorta and collected into a silicone tube, and then the tube was placed in a prewarmed MH3000 thrombosis instrument (Yimeikang, Zhengzhou, China). After 10 min of rotation, the thrombus was carefully pulled out from the tube. The liquid in the surface of the thrombus was removed lightly by filter paper. Wet thrombus weight and length were immediately determined [38].

#### 3.10.2. Carotid Artery Thrombosis In Vivo

Antithrombotic activity in vivo was performed using thrombosis instruments according to the method of Ulusal et al. [62]. Male SD rats were randomly divided into different experimental groups (12 rats/group). Rats were anaesthetized with 15% urethane and injected with MS-1 (2.5, 5, and 10 mg/kg), heparin (0.5 mg/kg) or saline solution. The right carotid artery was isolated, and then the artery was stimulated with 7.6 mA direct current for 7 min at the proximal end of the artery. The thrombus occlusion time was monitored by a temperature sensor until alarm, which was applied to the distal end of the carotid artery.

### 3.11. Statistical Analysis

The bioassay results were expressed as means ± standard deviation (SD). At least three samples were prepared for assays of every attribute. The experimental data were subjected to one-way ANOVA analysis with Turkey’s test (GraphPad Prism 7.00, La Jolla, CA, USA). Statistical significance is denoted by asterisks and hashes. * and # represented *p* < 0.05, while ** and ^##^ were *p* < 0.01.

## 4. Conclusions

MS-1 was a novel sulfated heterorhamnan with branches consisting of 4-linked β-d-xylose, 4-/6-linked d-glucose, terminal β-d-glucuronic acid, and 3-/2-linked α-l-rhamnose. MS-1 had strong anticoagulant activity in vivo and in vitro, and reduced platelet aggregation. The anticoagulant property of MS-1 was mainly attributed to powerful potentiation thrombin by HC-II, and it also hastened thrombin and coagulation factor Xa inhibitions mediated by AT-III. Moreover, MS-1 exhibited high thrombolytic and antithrombotic activities in vivo and in vitro. MS-1 had potential as a marine drug for prevention and therapy of thromboembolic disease. An in-depth investigation is underway to characterize the properties of this active MS-1. Continuous efforts will lead to developments of algal derived anticoagulant and antithrombotic agents for clinical uses.

## Figures and Tables

**Table 1:** Results of methylation analyses of Monostroma sulfated polysaccharide (MS-1) and Monostroma desulfated polysaccharide (DSMS-1).

Methylated Alditol Acetate	Linkage Pattern	Molar Percent Ratio	
		MS-1	DSMS-1
1,5-Di-*O*-acetyl-2,3,4-tri-*O*-methyl-rhamnitol	Rha*p*-(1→	3.53	3.91
1,5-Di-*O*-acetyl-2,3,4-tri-*O*-methyl-xylitol	Xyl*p*-(1→	0.68	0.62
1,2,5-Tri-*O*-acetyl-3,4-di-*O*-methyl-rhamnitol	→2)-Rha*p*-(1→	22.68	30.84
1,3,5-Tri-*O*-acetyl-2,4-di-*O*-methyl-rhamnitol	→3)-Rha*p*-(1→	30.02	45.17
1,5-Di-*O*-acetyl-2,3,4,6-tetra-*O*-methyl-glucitol	Glc*p*-(1→	1.03	1.13
1,4,5-Tri-*O*-acetyl-2,3-tri-*O*-methyl-xylitol	→4)-Xyl*p*-(1→	1.38	1.72
1,3,4,5-Tetra-*O*-acetyl-2-*O*-methyl-rhamnitol	→3,4)-Rha*p*-(1→	9.27	1.13
1,2,3,5-Tetra-*O*-acetyl-4-*O*-methyl-rhamnitol	→2,3)-Rha*p*-(1→	20.87	12.10
1,2,4,5-Tetra-*O*-acetyl-3-*O*-methyl-rhamnitol	→2,4)-Rha*p*-(1→	7.16	0.57
1,6,5-Tri-*O*-acetyl-2,3,4-tri-*O*-methyl-glucitol	→6)-Glc*p*-(1→	1.42	1.17
1,4,5-Tri-*O*-acetyl-2,3,6-tri-*O*-methyl-glucitol	→4)-Glc*p*-(1→	1.86	1.64

**Table 2:** ^1^H and ^13^C chemical shifts (ppm) of MS-1.

Rhamnosyl Residues		Chemical Shift (ppm)					
		1	2	3	4	5	6
A →3)-α-l-Rha*p*-(1→	^1^H	5.07	4.20	3.96	3.59	4.10	1.43
	^13^C	103.32	71.36	79.31	73.16	70.69	18.39
B →2)-α-l-Rha*p*-(1→	^1^H	5.23	4.28	4.00	3.59	4.14	1.38
	^13^C	101.85	79.78	70.72	73.19	70.79	18.39
C →2)-α-l-Rha*p*(4SO_4_)-(1→	^1^H	5.25	4.30	4.12	4.39	4.10	1.43
	^13^C	101.85	79.78	70.86	80.39	70.74	18.39
D →2,3)-α-l-Rha*p*-(1→	^1^H	5.39	4.26	4.09	3.60	4.00	1.38
	^13^C	101.31	79.30	80.46	73.19	70.67	18.39
E →3)-α-l-Rha*p*(4SO_4_)-(1→	^1^H	5.45	4.25	4.00	4.31	4.02	1.35
	^13^C	100.40	71.46	79.35	81.99	70.69	18.39
F →3)-α-l-Rha*p*(2SO_4_)-(1→	^1^H	5.50	4.70	4.11	3.58	3.98	1.35
	^13^C	100.76	78.18	77.42	73.16	70.51	18.39

**Table 3:** Result of anticoagulant activity assay of MS-1 in vitro.

Clotting Time ^a^ (s)	Sample	Concentration (μg/mL)				
		0	5	10	25	50
APTT	MS-1	40.3 ± 1.1	59.2 ± 0.5 *	97.8 ± 5.0 **	>200 **	-
	Heparin		127.8 ± 12.9 **	>200 **	-	-
TT	MS-1	14.5 ± 0.3	17.1 ± 0.2	19.4 ± 1.5 *	57.4 ± 3.6 **	>120 **
	Heparin		70.3 ± 0.9 **	>120 **	-	-
PT	MS-1	13.9 ± 0.7	12.8 ± 0.1	13.3 ± 0.2	15.5 ± 0.5	19.3 ± 0.8
	Heparin		17.4 ± 4.4 *	33.6 ± 1.7 **	81.3 ± 2.2 **	>120 **

^a^ All data were the mean of three parallel assays and expressed as means ± standard deviation (SD). * *p* < 0.05, ** *p* < 0.01, versus the control group.

## Supplementary Materials

The following are available online at link https://www.mdpi.com/1660-3397/17/4/247/s1. Figure S1: HPGPC chromatogram, HPLC chromatogram and IR spectrum of MS-1, Figure S2: NMR spectra of MS-1, Figure S3: HILIC-FTMS profiling and HPLC chromatogram of MS-1-O, Figure S4: Negative-ion ES-CID-MS/MS/MS product–ion spectra and assignments of the ions. (A) ES-MS spectrum of ion at *m*/*z* 339; (B) ES-MS spectrum of ion at *m*/*z* 419; (C) ES-MS spectrum of ion at *m*/*z* 565.
