# Supplementary material for: Anticoagulant and Antithrombotic Properties in Vitro and in Vivo of a Novel Sulfated Polysaccharide from Marine Green Alga Monostroma nitidum
Source: Mar Drugs. 2019 Apr 25;17(4):247. doi: 10.3390/md17040247 (PMC6521212; doi:10.3390/md17040247)
Supplement: Supplementary file 1 [file marinedrugs-17-00247-s001.pdf]

SUPPLEMENTARY TABLES

Table S1. ^1H and ^{13}C chemical shifts data of DSMS-1.

Rhamnosyl residues	Nucleus	Chemical shift (ppm)					
		1	2	3	4	5	6
A→3)-α-L-Rha*p*-(1→	^1H	5.00	4.20	3.89	3.60	3.91	1.38
	^{13}C	103.55	71.41	79.02	72.28	70.73	18.32
B→3)-α-L-Rha*p*-(1→	^1H	5.07	4.18	3.89	3.60	3.91	1.38
	^{13}C	103.72	71.41	79.02	72.28	70.73	18.32
C→2)-α-L-Rha*p*-(1→	^1H	5.23	4.11	3.93	3.56	3.98	1.35
	^{13}C	102.41	79.40	70.78	73.16	71.43	18.32
D→2)-α-L-Rha*p*-(1→	^1H	5.30	4.11	3.93	3.56	3.98	1.35
	^{13}C	102.41	79.40	70.78	73.16	70.73	18.32
E→2,3)-α-L-Rha*p*-(1→	^1H	5.39	4.27	3.97	3.55	3.94	1.35
	^{13}C	102.37	80.90	79.00	73.29	71.43	18.32

SUPPLEMENTARY FIGURE LEGENDS

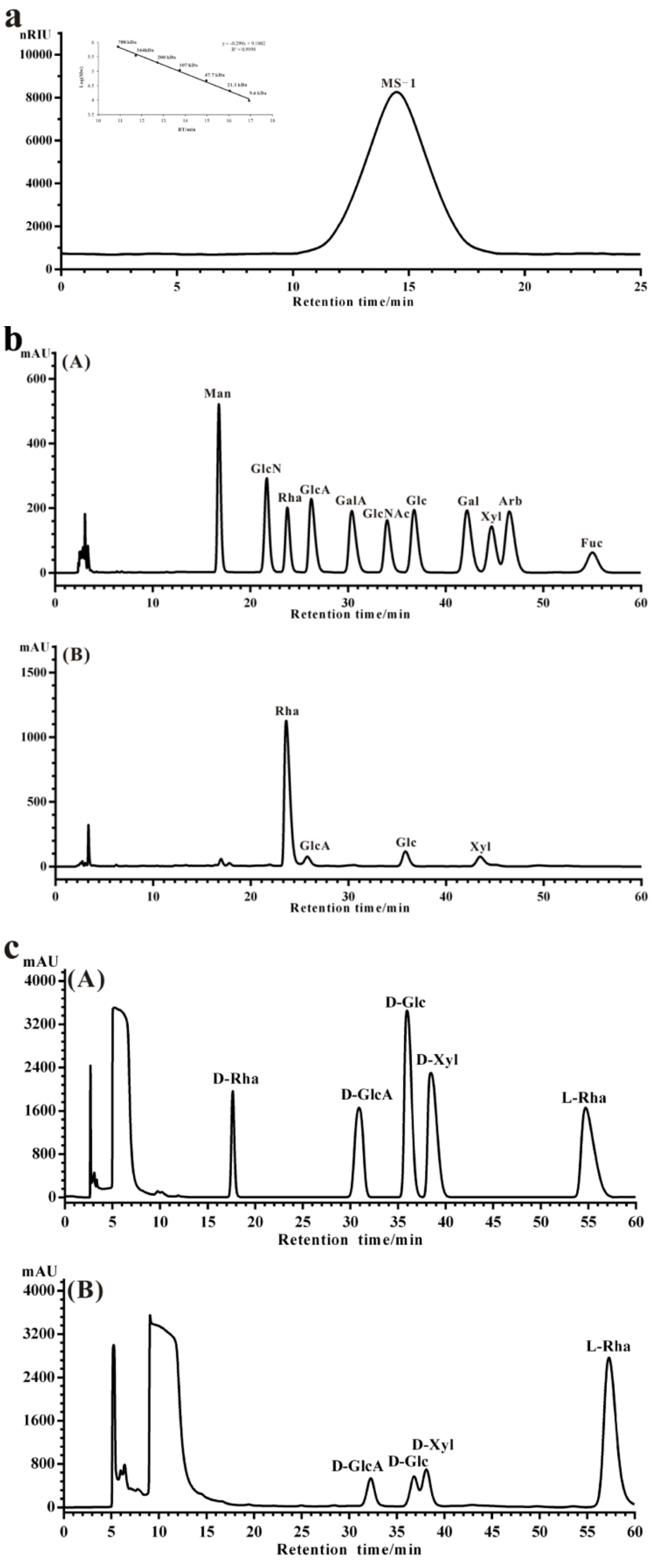

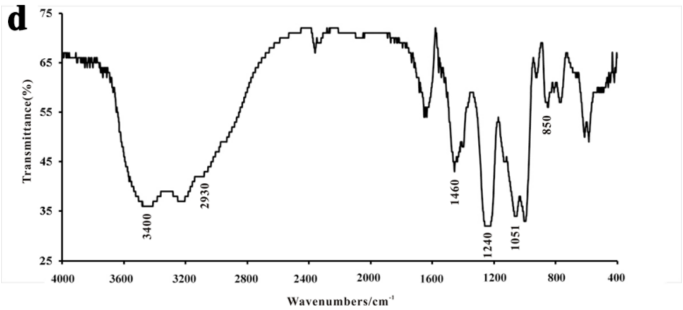

Figure S1. HPGPC chromatogram, HPLC chromatogram and IR spectrum of MS-1. (a) HPGPC chromatogram of MS-1 on a Shodex OHpak SB-804 HQ column and the standard curve of molecular weight; (b) HPLC chromatogram for monosaccharide composition analysis of MS-1 (Man: D-mannose, GlcN: D-glucosamine, Rha: L-rhamnose, GlcA: D-glucuronic acid, GalA: D-galacturonic acid, Glc: D-glucose, Gal: D-galactose, Xyl: D-xylose, Ara: L-arabinose, Fuc: L-fucose); (c) HPLC chromatogram of the sugar configuration determination of MS-1 (D-Rha: D-rhamnose, D-GlcA: D-glucuronic acid, D-Glc: D-glucose, D-Xyl: D-xylose, L-Rha: L-rhamnose); (d) IR spectrum of MS-1.

(A)

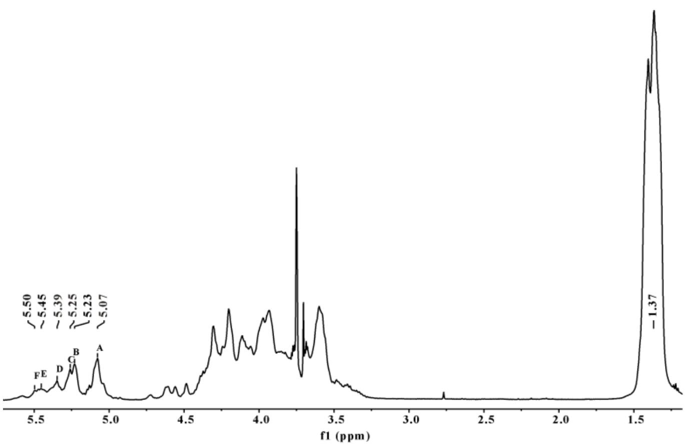

(B)

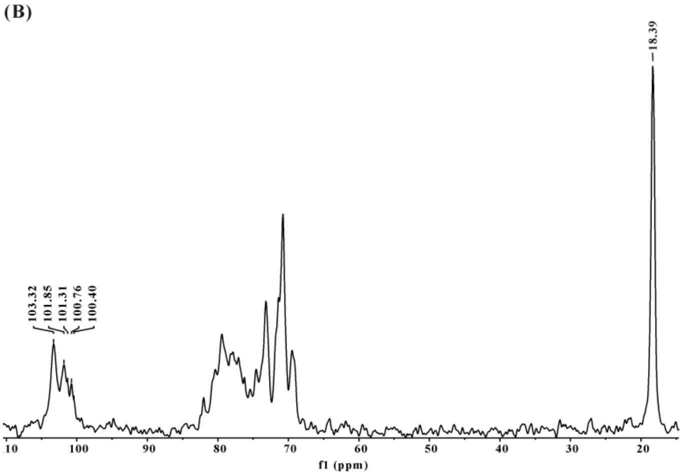

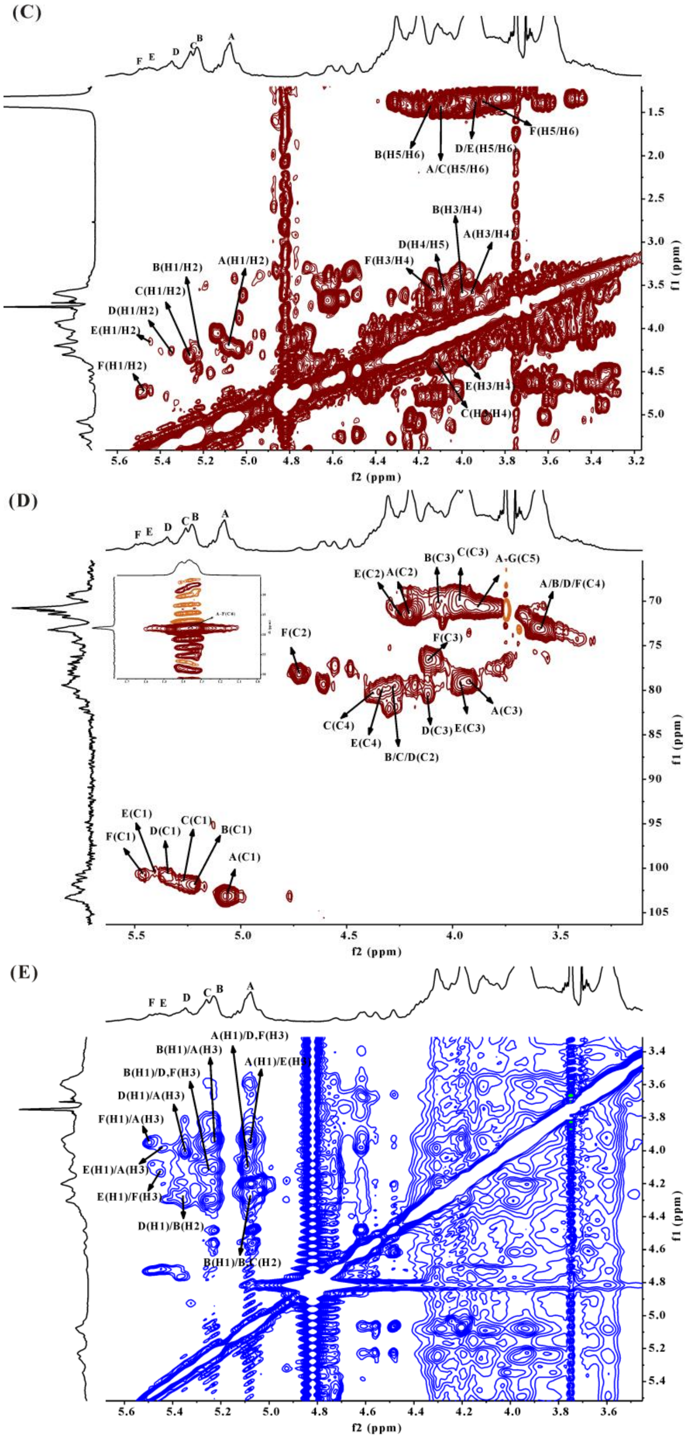

Figure S2. NMR spectra of MS-1. Spectra were performed on an Agilent DD2 500M NMR spectrometer. Chemical shifts are referenced to internal acetone at 2.225 ppm for ^{1}H and 31.07 ppm for ^{13}C. (A) ^{1}H NMR spectrum; (B) ^{13}C NMR spectrum; (C) ^{1}H–^{1}H COSY spectrum; (D) ^{1}H–^{13}C HSQC spectrum; (E) ^{1}H–^{1}H NOESY spectrum. A–F correspond to →3)-α-L-Rha*p*-(1→, →2)-α-L-Rha*p*-(1→, →2)-α-L-Rha*p*(4SO_4)-(1→, →2,3)-α-L-Rha*p*-(1→, →3)-α-L-Rha*p*(4SO_4)-(1→ and →3)-α-L-Rha*p*(2SO_4)-(1→, respectively. Rha*p*: rhamnopyranose.

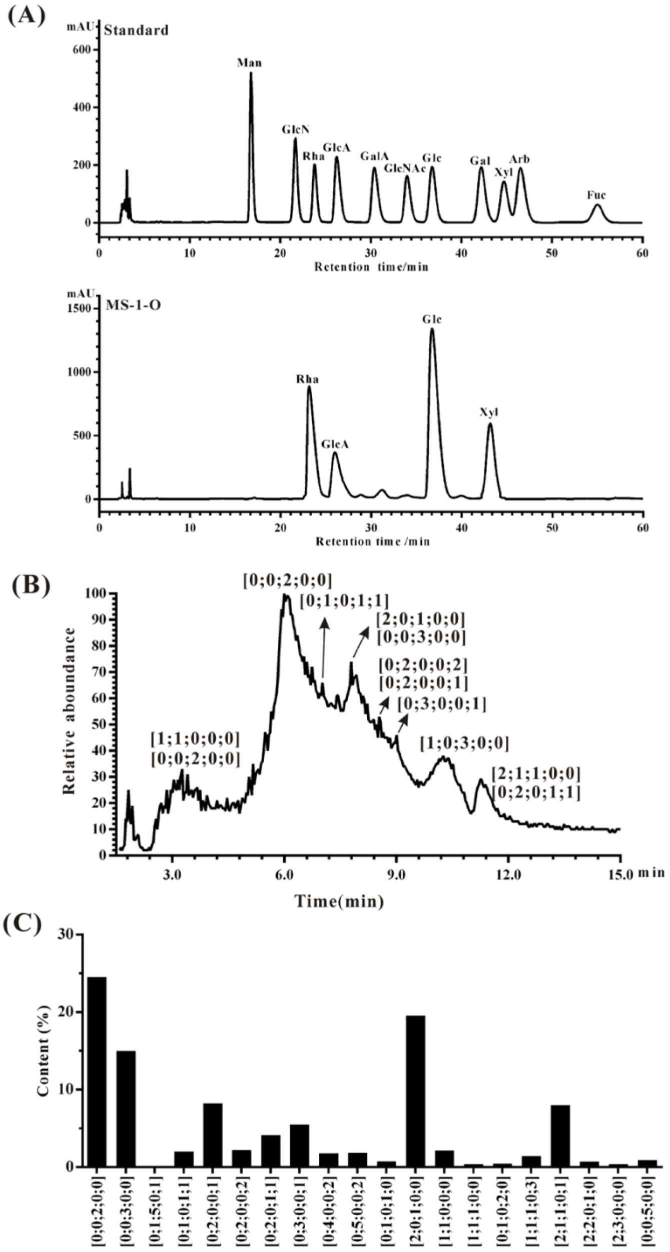

Figure S3. HILIC-FTMS profiling and HPLC chromatogram of MS-1-O. (A) HPLC chromatogram for monosaccharide composition analysis of MS-1-O (Man: D-mannose, GlcN: D-glucosamine, Rha: L-rhamnose, GlcA: D-glucuronic acid, GalA: D-galacturonic acid, Glc: D-glucose, Gal: D-galactose, Xyl: D-xylose, Ara: L-arabinose, Fuc: L-fucose); (B) total ion chromatography of MS-1-O; (C) composition analysis of MS-1-O calculated by GlycResoft. The analytical error for each oligosaccharide was < 5%. Oligomer composition was given as [Xyl; Rha; Glc; GlcA; SO_3].

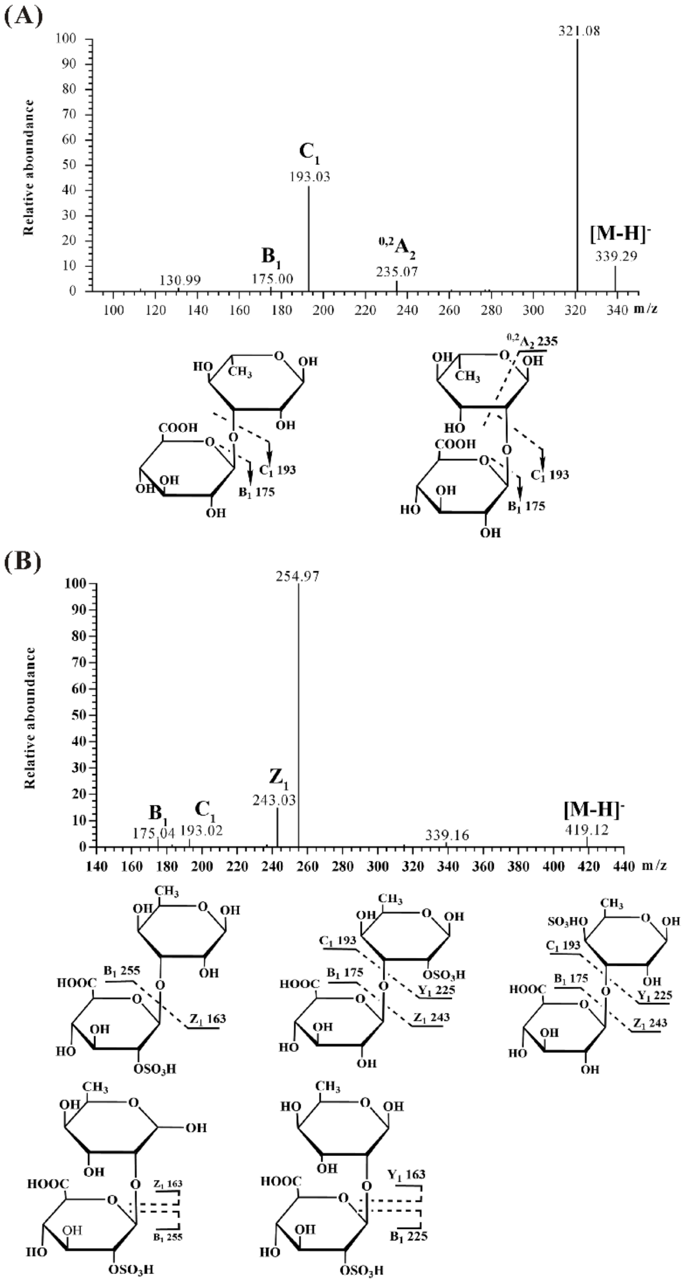
(A)
Relative abondance
m/z
321.08
C1
193.03
B1
175.00
130.99
0,2A2
235.07
[M-H]-
339.29
C1 193
B1 175
0,2A2 235
(B)
Relative abondance
m/z
254.97
Z1
243.03
B1
175.04
C1
193.02
339.16
[M-H]-
419.12
B1 255
Z1 163
C1 193
B1 175
Y1 225
Z1 243
Z1 163
B1 255
Y1 163
B1 225

(C)

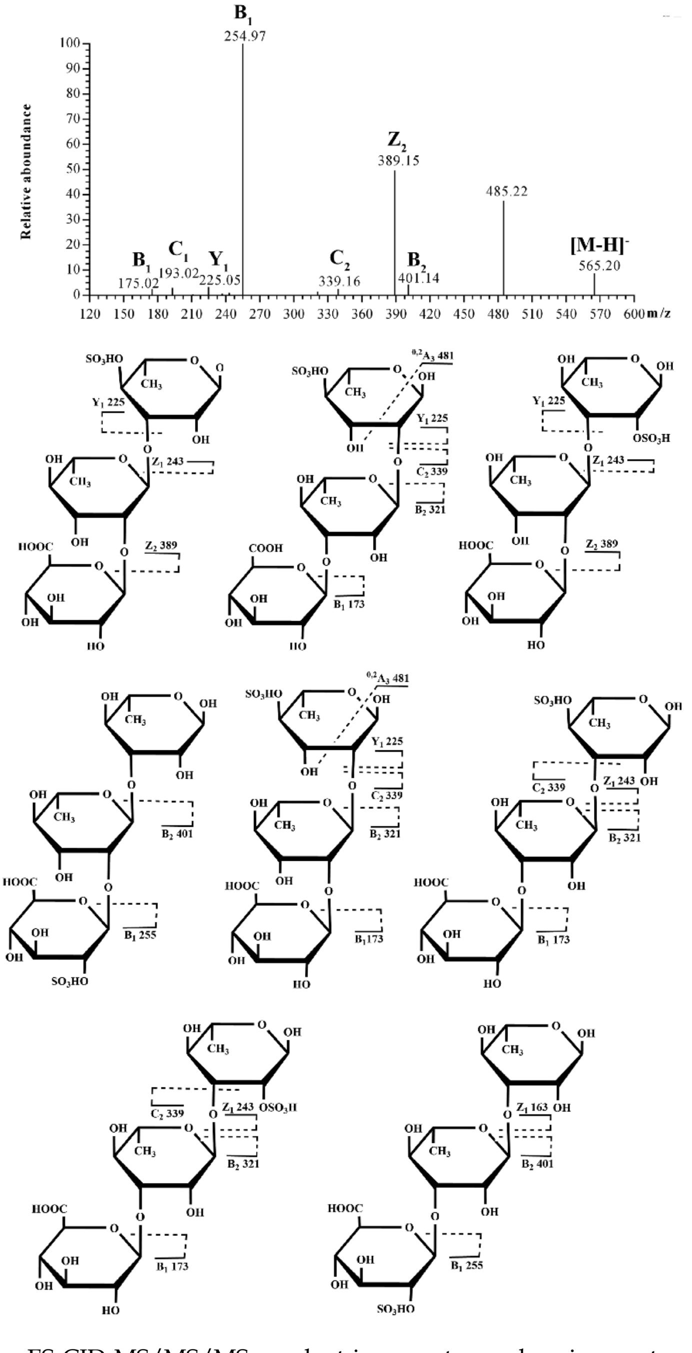

Figure S4. Negative-ion ES-CID-MS/MS/MS product-ion spectra and assignments of the ions. (A) ES-MS spectrum of ion at *m/z* 339; (B) ES-MS spectrum of ion at *m/z* 419; (C) ES-MS spectrum of ion at *m/z* 565.
